# Proprotein Convertase Subtilisin/Kexin Type 9 (PCSK9) Inhibitors: A Novel Therapy in the Treatment of Cholesterol Crystal Embolism-Induced Kidney Injury

**DOI:** 10.1016/j.xkme.2025.101125

**Published:** 2025-09-19

**Authors:** Benjamin J. Strimaitis, Leo Francis, Eoin D. O’Sullivan

**Affiliations:** 1 Kidney Health Service, Metro North Hospital and Health Services, Brisbane, QLD, Australia; 2 Department of Anatomical Pathology, Royal Brisbane and Women's Hospital, Brisbane, QLD, Australia; 3 Institute of Molecular Bioscience, University of Queensland, Brisbane, QLD, Australia; 4 QIMR Berghofer Medical Research Institute, Brisbane, QLD, Australia

**Keywords:** Acute kidney injury, cholesterol emboli, eosinophilia, PCSK9 inhibitors

## Abstract

Cholesterol crystal embolism (CCE) is a challenging and often underrecognized cause of kidney impairment, especially in elderly patients with significant atherosclerotic disease. CCE typically arises from dislodged cholesterol crystals that migrate from atherosclerotic plaques to occlude small renal and systemic arteries. Here, we report a case of a 72-year-old man who presented with acute kidney injury following coronary artery bypass graft surgery. Five weeks postsurgery the patient experienced a rapid decline in kidney function. Kidney biopsy confirmed the diagnosis of CCE, showing cholesterol clefts and associated inflammation within renal arteries. Although the patient initially responded to high-dose corticosteroid therapy kidney function declined during steroid tapering. A proprotein convertase subtilisin/kexin type 9 (PCSK9) inhibitor, evolocumab, was introduced as an adjunct treatment, targeting lipid reduction and plaque stabilization. Following initiation of evolocumab, kidney function stabilized and gradually improved, with concurrent reductions in eosinophilia, suggesting a decrease in ongoing embolic phenomena. This case illustrates the potential role of PCSK9 inhibitors as an adjunctive therapy for CCE-associated kidney injury, particularly when conventional management may be insufficient. PCSK9 inhibitors warrant further investigation in CCE cases with kidney involvement, as they may offer additional benefits beyond lipid lowering by reducing plaque instability and associated microinflammation.

Cholesterol crystal embolism (CCE) is an often underdiagnosed cause of kidney injury, particularly in elderly patients with advanced vascular disease.^1^, ^2^, ^3^ This condition arises from showers of cholesterol crystals that dislodge from atherosclerotic plaques, typically in the aorta, and occlude small renal arteries. Although spontaneous cases occur, cholesterol crystal embolization is often triggered by invasive vascular procedures, such as angiography, vascular surgery, or the administration of anticoagulant or fibrinolytic therapies.^4^^,^^5^ Clinically, kidney outcomes are variable. Some patients experience mild kidney impairment, whereas others progress to severe kidney failure, requiring dialysis. Acute presentations may manifest as sudden kidney failure, but more commonly kidney function declines progressively over weeks or remains chronically impaired in a stable state.

Here, we describe a case of subacute CCE-induced acute kidney injury rescued with evolocumab, a novel proprotein convertase subtilisin/kexin type 9 (PCSK9) inhibitor.

## Patient Information

A 72-year-old man was admitted for evaluation of a marked decline in kidney function over an 8-week period. The patient had suffered an ST-elevation myocardial infarction and underwent subsequent emergency coronary bypass grafting before the decline in kidney function. At discharge following his surgery, his estimated glomerular filtration rate (eGFR) had been recorded at 44 mL/min/1.73 m^2^ (creatinine 137 μmol/L, 1.55 mg/dL) and had been reduced to 13 mL/min/1.73 m^2^ (creatinine 372 μmol/L, 4.21 mg/dL) over the 8 weeks since surgery and before admission.

His key medical history was significant for a ST-elevation myocardial infarction in the 5 weeks before admission, which had been treated with a triple coronary bypass grafting involving left internal mammary artery to left anterior descending artery, saphenous vein graft to obtuse marginal, and saphenous vein graft to posterior descending artery. Additional history included gout, type 2 diabetes mellitus (HbA1c 5.9% 6 months before admission), secondary hyperparathyroidism, and a twenty-pack year history of smoking.

His medications included 80 mg atorvastatin daily, 5 mg amlodipine daily, 75 mg clopidogrel daily, 2.5 mg bisoprolol daily, 10 mg dapagliflozin daily, 5 mg linagliptin daily, and 1,500 mg calcium/12.5 mg of vitamin D daily.

On examination he was euvolemic, with a blood pressure of 160/90 mm Hg. No rashes, cardiovascular abnormalities, or distal vascular abnormalities were noted. Key results are summarized in Table 1.

**Table 1:** Key Presenting Results

Test	Result	Normal Range
Blood tests		
White blood cell, x10^9^/L	7.2	3.5-11.0
Hemoglobin, g/L	108	120-180
Platelets, x10^9^/L	217	140-400
Eosinophils x10^9^/L	0.53	<0.40
Sodium, mmol/L	140	135-145
Potassium, mmol/L	5.4	3.5-5.2
Urea, mmol/L	26.6 (74.4 mg/dL)	2.9-8.2 (5-10 mg/dL)
Creatinine, μmol/l	372 (4.21 mg/dL)	64-108, (0.6-1.1 mg/dL)
eGFR, mL/min/1.73 m^2^	13	>90
Fasting total cholesterol, mmol/L	2.4 mmol/L (92.8 mg/dL)	3.6-7.3 (<200 mg/dL)
Trig, mmol/L	1.3 (115 mg/dL)	<2.0 (<150 mg/dL)
HDL, mmol/L	0.77 (27 mg/dL)	>1.0 (>60 mg/dL)
LDL mmol/L	1.04 (40 mg/dL)	<2.5 (<200 mg/dL)
Albumin, g/L	45	35-50
Hep B, HEP C, HIV screen	Negative	-
CRP, mg/L	15	<5.0
ANA	1:80 nucleolar pattern	-
ANCA, ENA, anti-GBM, C3, C4	Normal	-
Serum electrophoresis	Normal	-
Urine tests		
Urinalyses	Protein +, Glucose ++, no leukocytes, erythrocytes, eosinophils	-
Albumin creatinine ratio g/mol creatinine	0.6	<2.5 g/mmol
Urinary Bence Jones proteins	Negative	-

SI units are used, with conventional units (eg, mg/dL) provided in parentheses when relevant.

Abbreviations: ANA, antinuclear antibody; ANCA, anti-neutrophil cytoplasmic antibody; anti-GBM, anti-glomerular basement membrane antibody; C3/C4, complement components 3 and 4; CRP, C-reactive protein; eGFR, estimated glomerular filtration rate; ENA, extractable nuclear antigen; HDL, high-density lipoprotein; LDL, low-density lipoprotein; Trig, triglycerides.

**Figure 1:**
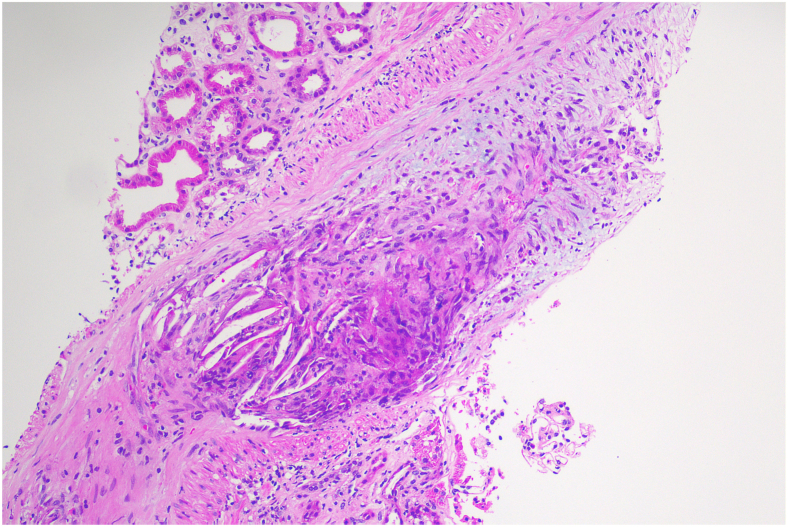
This interlobular artery, viewed in longitudinal section, shows occlusion of its lumen by multinucleated giant cells admixed with cholesterol clefts. These clear spaces represent spaces in which cholesterol crystals were dissolved during the processing of the biopsy sample. The tunica intima also shows loose fibrointimal hyperplasia (H&E stain). Abbreviation: H&E, hematoxylin and eosin.

A kidney biopsy showed atheromatous embolism occluding at least one interlobular artery, with luminal obstruction because of the presence of cholesterol clefts and an associated histiocytic reaction, increased globally sclerosed glomeruli (32% of biopsy), and normal immunofluorescence results. There was tubular atrophy and interstitial fibrosis involving approximately 20% of the cortex. Electron microscopy was not performed (Fig 1).

A 24-hour Holter did not demonstrate any arrhythmia. An echocardiogram did not show a thrombus or embolic source and all valves including aortic valve were normal. A computed tomography aorto-abdominal vessel angiogram showed significant burden of atheromatous disease in the thoracic and abdominal aorta. Magnetic resonance imaging of abdominal vessels confirmed moderate atheromatous disease through the arterial tree with preserved flow voids in the major branches of the abdominal aorta with no blockage of the renal arteries.

**Figure 2:**
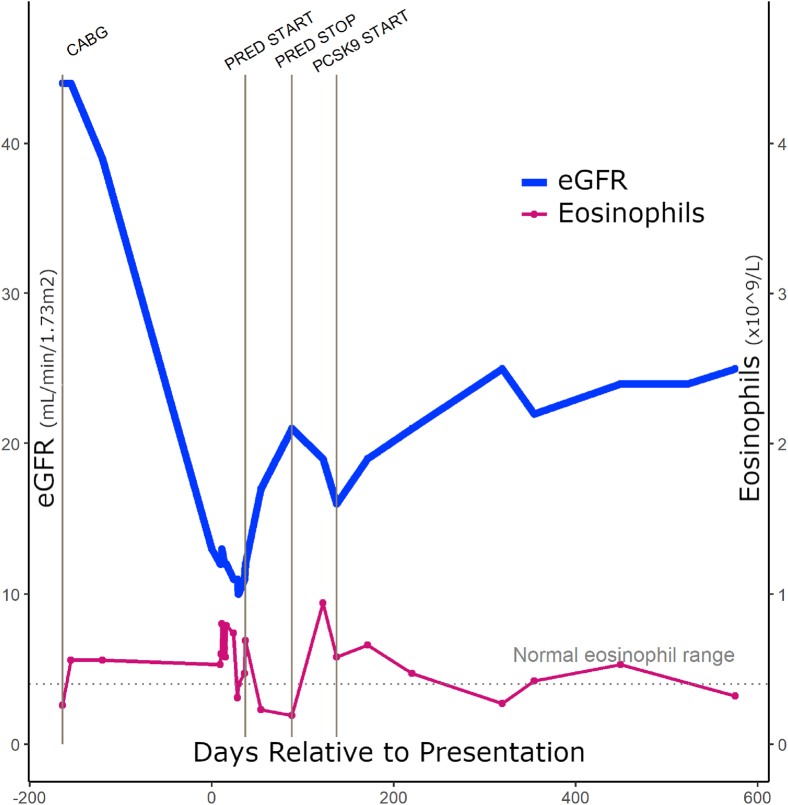
Timeline of estimated glomerular filtration rate (eGFR) and eosinophil counts in a patient with PCSK9 inhibition treatment. The plot shows the changes in eGFR and eosinophil levels over time, relative to presentation (x axis, in days). eGFR (in mL/min/1.73 m^2^) is represented by the blue line and points, whereas eosinophil levels (×10^9^/L) are shown on the secondary y axis with a pink line and points. Event markers and corresponding labels black text) are shown at specific time points, indicating relevant clinical events. Abbreviations: CABG, coronary artery bypass graft; PCSK9, proprotein convertase subtilisin/kexin type 9; PRED START/STOP, commencement and cessation of oral prednisolone.

Considering the degree of peri-embolic inflammation on the biopsy, 50 mg prednisolone daily was started with a rapid wean over 6 weeks alongside aggressive risk factor control (vegetarian and very low salt diet). Blood pressure was <120/70 mm Hg, low-density lipoprotein (LDL) was maintained at 1.36 mmol/L (52.5 mg/dL) and high-density lipoprotein at 0.99 mmol/L (38.2 mg/dL). A serum lipoprotein(a) level of 158 nmol/L (73.4 mg/dL) was noted (normal range: <30 mg/dL or <75 nmol/L). The patient suffered hyperglycemia, insomnia, and mild agitation on steroids. The kidney function began to deteriorate alongside a marked increase in eosinophilia during the steroid wean (Fig 2).

To further stabilize the atheromatous plaques, evolocumab was started at 140 mg subcutaneously every 2 weeks resulting in stabilization of kidney function. Over time, the kidney function has continued to improve alongside a reduction in serum eosinophils, suggesting a reduction in emboli phenomena (Fig 2). The patient continued to receive fortnightly evolocumab with plans to continue indefinitely; the most recent eGFR 22 months after initiation was stable at 23 mL/min/1.72 m^2^.

## Discussion

Kidney injury post CCE is typically diagnosed via kidney biopsy which shows an organizing atheroembolus made up of cholesterol clefts, macrophages, and lymphocytes, showing changes consistent with either focal segmental glomerulosclerosis, interstitial fibrosis and tubular atrophy.^3^ Supporting laboratory findings include elevated serum creatinine, microscopic hematuria, minimal proteinuria, and eosinophilia either in the urine or blood.^6^

The inflammatory response around occluded vessels contributes to ongoing kidney damage in CCE, leading to progressive fibrosis and glomerulosclerosis. Microinflammation observed in CCE is typically driven by macrophages and lymphocytes reacting to cholesterol clefts. Reducing this inflammation has been a therapeutic challenge in managing CCE, as shown by the patient’s initial response to corticosteroids, which provided transient kidney improvement but led to adverse effects like hyperglycemia and agitation.

The treatment of CCE kidney disease has until recently consisted of supportive management in the acute phase and cessation of anticoagulation when appropriate.^7^^,^^8^ Longer term risk factor modification with antihypertensive and lipid-lowering therapies is common practice; however, these have not been shown to reverse the disease process.^6^ Corticosteroids have been used with the aim of reducing the microreactive inflammatory response of these cholesterol emboli.^4^^,^^5^^,^^9^ However, long-term corticosteroids are problematic in many patients because of the well documented side effects of steroid use.

**Figure 3:**
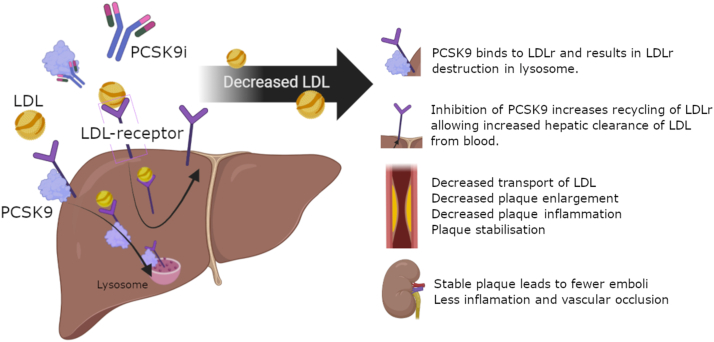
Mechanisms of PCSK9 inhibition’s potential benefit in CCE. CCE, cholesterol crystal embolism; PCSK9, proprotein convertase subtilisin/kexin type 9.

PCSK9 inhibitors are a new lipid-lowering agent which may be a novel treatment option for patients with CCE-induced kidney injury. PCKS9 inhibitors, such as evolocumab, are a human monoclonal antibody that targets PCSK9.^10^ Inhibiting PCSK9 reduces the recycling of LDL receptors in the liver, drastically increasing LDL cholesterol clearance which is the main precursor of atherosclerotic plaques.^11^ Furthermore, PCSK9 inhibitors reduce inflammatory cytokines and promotes atherosclerotic plaque regression (Fig 3).^12^ Recent trials have found PSCK9 inhibitors to be safe and efficacious at levels of kidney function as low as eGFR 20 mL/min/1.73 m^2^ and case reports are emerging of safe use in kidney transplant.^13^, ^14^, ^15^ In a 2022 case report, a 70-year-old woman with acute kidney injury following coronary intervention showed stabilization of her eGFR at 30 mL/min/1.73 m^2^ over 52 weeks following PCSK9i therapy.^10^ Similarly, an eGFR improvement from 9 to 18 mL/min/1.73 m^2^ was reported in a 77-year-old man with CCE following carotid stenting over 25 weeks with PCSK9i treatment.^16^

In the United States, PCSK9 inhibitors are Food and Drug Administration-approved for a range of indications including familial hypercholesterolemia (both heterozygous and homozygous forms), clinical atherosclerotic cardiovascular disease and as adjuncts in patients unable to reach LDL-C targets despite maximally tolerated statins and ezetimibe. American guidelines suggest PCSK9 inhibitors for patients at very high cardiovascular risk who are on maximally tolerated statin therapy and have an LDL-C level of 70 mg/dL (1.8 mmol/L) or higher. European guidelines support a risk-based approach in selected cases. Pooled trial data show that PCSK9 inhibition confers cardiovascular benefit even at lower LDL-C levels, with no clear lower threshold for benefit.^17^

The use of Lp(a) measurement was as an additional risk stratification tool used during clinical decision making and helped justify the use of PSCK9 inhibition specifically. Although Lp(a) levels can be high in kidney disease for several reasons, it may be potentially a major risk factor in these populations.^18^ Although risk thresholds for Lp(a) are uncertain, <30 mg/dL (or <75 nmol/L) is considered normal, 30-50 mg/dL (or 50-125 nmol/L) intermediate, and >50 mg/dL (or >125 nmol/L) abnormal in the European Atherosclerotic Society consensus statement. With a Lp(a) level of 158 nmol/L this patient was considered high risk. PCKS9 treatment is the only currently approved treatment demonstrated to have reduced Lp(a) in clinical trials, including the ODESSY OUTCOMES and FOURIER trials, which showed PCSK9 therapy can reduce Lp(a) levels by 23% to 27% and reduced cardiovascular outcomes.^19^^,^^20^ Thus, the patient’s higher Lp(a) level informed selection of PCSK9 inhibition as therapy to potentially provide additional cardiovascular risk reduction.

In this context, off-label use of evolocumab was initiated following multidisciplinary discussion and hospital-based approval. This was justified on clinical grounds given the patient's ongoing ischemic injury, lack of alternative disease-modifying therapies and considered in the context of the personal and economic impact of progression to dialysis. This approach aligns with evolving international guidance that supports judicious use in very high-risk individuals, particularly when conventional therapies have failed or are insufficient. We found serum eosinophils tracked with treatment and presumed ongoing embolization and warrant further investigation as potentially useful clinical measurement when titrating treatment.

It is important to acknowledge that this case report cannot establish causality between PCSK9 inhibition and kidney function recovery. The observed stabilization and subsequent improvement in kidney function may have occurred independently of evolocumab initiation, particularly given the natural history of CCE, which in some cases may plateau or partially recover once embolization ceases and inflammation subsides.^2^^,^^6^

This case highlights the potential of PCSK9 inhibitors as an adjunctive therapy for managing CCE in patients with kidney impairment. By targeting both lipid levels and inflammation, evolocumab was associated with a favorable outcome in a patient for whom standard management options were limited.

## References

[1] Fine M.J., Kapoor W., Falanga V. (1987). Cholesterol crystal embolization: a review of 221 cases in the English literature. Angiology.

[2] Scolari F., Tardanico R. (2000). Cholesterol crystal embolism: A recognizable cause of renal disease. Am J Kidney Dis.

[3] Lusco M.A., Najafian B., Alpers C.E., Fogo A.B. (2016). AJKD Atlas of Renal Pathology: Cholesterol Emboli. Am J Kidney Dis.

[4] Koga J., Ohno M., Okamoto K. (2005). Cholesterol embolization treated with corticosteroids: two case reports. Angiology.

[5] Masuda J., Tanigawa T., Nakamori S. (2013). Use of corticosteroids in the treatment of cholesterol crystal embolism after cardiac catheterization: a report of four Japanese cases. Intern Med.

[6] Meyrier A. (2006). Cholesterol crystal embolism: diagnosis and treatment. Kidney Int.

[7] Varis J., Kuusniemi K., Järveläinen H. (2010). Cholesterol microembolization syndrome: a complication of anticoagulant therapy. CMAJ.

[8] Igarashi Y., Akimoto T., Kobayashi T. (2017). Performing anticoagulation: a puzzling case of cholesterol embolization syndrome. Clin Med Insights Case Rep.

[9] Nakayama M., Nagata M., Hirano T. (2006). Low-dose prednisolone ameliorates acute renal failure caused by cholesterol crystal embolism. Clin Nephrol.

[10] Tomoi Y., Soga Y., Imada K. (2022). Use of proprotein converse subtilisin/kexin type 9 inhibitor to treat cholesterol crystal embolisms after catheterization: a report of three cases. Intern Med.

[11] Page M.M., Watts G.F. (2016). PCSK9 inhibitors – mechanisms of action. Aust Prescr.

[12] Tang Z.H., Peng J., Ren Z. (2017). New role of PCSK9 in atherosclerotic inflammation promotion involving the TLR4/NF-κB pathway. Atherosclerosis.

[13] Charytan D.M., Sabatine M.S., Pedersen T.R. (2019). Efficacy and safety of evolocumab in chronic kidney disease in the FOURIER trial. J Am Coll Cardiol.

[14] García-Agudo R., Rojas-Fernández M.Á., Canllavi-Fiel E., Proy-Vega B., Tejera-Muñoz A. (2023). Safe and successful treatment with PCSK9 inhibitors in hypercholesterolemia and renal transplantation: a case report. Transplant Proc.

[15] Igweonu-Nwakile E.O., Ali S., Paul S. (2022). A systematic review on the safety and efficacy of PCSK9 inhibitors in lowering cardiovascular risks in patients with chronic kidney disease. Cureus.

[16] Morino J., Hirai K., Kaneko S. (2020). Successful treatment of cholesterol crystal embolism with anti-proprotein convertase subtilisin/kexin type 9 (PCSK9) antibody: a case report. Ren Fail.

[17] Mach F., Baigent C., Catapano A.L. (2020). 2019 ESC/EAS guidelines for the management of dyslipidaemias: lipid modification to reduce cardiovascular risk: the task force for the management of dyslipidaemias of the European Society of Cardiology (ESC) and European Atherosclerosis Society (EAS). Eur Heart J.

[18] Kronenberg F. (2014). Causes and consequences of lipoprotein(a) abnormalities in kidney disease. Clin Exp Nephrol.

[19] Schwartz G.G., Steg P.G., Szarek M. (2018). Alirocumab and cardiovascular outcomes after acute coronary syndrome. N Engl J Med.

[20] Sabatine M.S., Giugliano R.P., Keech A.C. (2017). Evolocumab and clinical outcomes in patients with cardiovascular disease. N Engl J Med.

